# Skipping Breakfast and the Risk of Cardiovascular Disease and Death: A Systematic Review of Prospective Cohort Studies in Primary Prevention Settings

**DOI:** 10.3390/jcdd6030030

**Published:** 2019-08-22

**Authors:** Richard Ofori-Asenso, Alice J. Owen, Danny Liew

**Affiliations:** Department of Epidemiology and Preventive Medicine, Monash University, Melbourne, Victoria, VIC 3004, Australia

**Keywords:** breakfast, meal frequency, cardiovascular disease, mortality

## Abstract

Several studies have associated skipping (not having) breakfast with cardiometabolic risk factors such as obesity, high blood pressure, unfavorable lipid profiles, diabetes, and metabolic syndrome. We examined the available evidence regarding the effect of skipping breakfast on cardiovascular morbidity and mortality, as well as all-cause mortality. Medline, Embase, and Web of Science were searched from inception until May 2019 to identify prospective cohort studies that examined the association between skipping breakfast and the risk of cardiovascular morbidity and mortality and all-cause death. Electronic searches were supplemented by manual screening of the references of retrieved studies. Out of 456 citations identified, four studies (from Japan and the US) were included. The included studies involved a total of 199,634 adults (aged ≥40 years; 48.5% female) without known cardiovascular disease (CVD) at baseline followed over a median duration of 17.4 years. The pooled data suggested that people who regularly skipped breakfast were about 21% more likely (hazard ratio (HR) 1.21, 95% confidence interval (CI) 1.08–1.35; *I*^2^ = 17.3%, *p* = 0.304) to experience incident CVD or die from it than people who regularly consumed breakfast. Also, the risk of all-cause death was 32% higher (HR 1.32, 95% CI 1.17–1.48; *I*^2^ = 7.6%, *p* = 0.339) in people who regularly skipped breakfast than in people who regularly consumed breakfast. However, the definition of skipping breakfast was heterogenous and adjustment for confounders varied significantly. Therefore, residual confounding could not be ruled out and caution is required in the interpretation of the findings. Hence, large prospective studies with a consistent definition of skipping breakfast, and conducted across different populations, are needed to provide more robust evidence of the health effects of skipping breakfast.

## 1. Background

Cardiovascular diseases (CVDs) remain the leading contributors to disease burden and deaths worldwide. In 2015, there were more than 422 million cases of CVD, resulting in over 17.9 million deaths (~31% of all global deaths) [1]. Much of CVD is preventable through being physically active, not smoking, and consuming a healthy diet [2,3].

Whilst evidence-based recommendations relative to eating habits (timing, quantity, energy content, and frequency) in adults are lacking in most nutritional guidelines [4,5], for many decades breakfast has been touted as the most important meal of the day [6]. Indeed, some have suggested that approximately 15–30% of daily energy intake should be consumed at breakfast [7,8]. Yet, more than a quarter of adults skip this presumably most important meal of the day [9,10,11]. Skipping breakfast is notably common in socioeconomically disadvantaged people, shift workers or those with excessive working hours, as well as people with disinterest in food (such as those with depression), and those with poor health literacy [12,13,14].

Epidemiological studies have demonstrated a higher prevalence and incidence of cardiometabolic risk factors such as obesity, high blood pressure, unfavorable lipid profiles, diabetes, and metabolic syndrome in people who skip breakfast, which may ultimately contribute to increased risk of CVD [15,16,17]. Recently, Uzhova and colleagues reported that, compared with consuming a high-energy breakfast, regularly skipping breakfast was associated with a 1.6- and 2.6-fold higher likelihood of non-coronary and generalized atherosclerosis, respectively, after controlling for conventional cardiovascular risk factors and diet quality [18].

However, the exact magnitude of the effect of skipping breakfast on the risk of cardiovascular morbidity and mortality, and all-cause death, remains poorly understood. Improved understanding of the impact of skipping breakfast on the onset of CVD and related death may help to inform guideline development and public health interventions. Thus, we conducted a systematic review to examine the available evidence regarding the effect of skipping breakfast on the risk of incident CVD and mortality, as well as all-cause death, in the primary prevention setting.

## 2. Methods

This systematic review followed the methods outlined by the Cochrane collaboration, as well as the Guidelines of the Preferred Reporting for Systematic Reviews and Meta-Analyses (PRISMA) statement [19].

### 2.1. Search Strategy and Study Selection

We searched Medline, Embase, and Web of Science from inception until 5 May 2019 for studies published in English that reported the prospective association between skipping breakfast and the risk of CVD development and mortality as well as all-cause death. The following search terms were used in combination: (“cardiovascular disease” OR “cardiovascular disorders” OR “coronary heart disease” OR “coronary artery disease” OR “acute coronary syndrome” OR “ischemic heart disease” OR “myocardial infarction” OR “myocardial ischemia” OR “heart attack” OR “heart failure” OR “stroke” or “cerebrovascular disease” OR “cardiac arrest” OR “unstable angina” OR “mortality” OR “death”) AND (“breakfast” OR “breakfast skipping” OR “skipping breakfast” OR “morning meal” OR “meal skipping” OR “meal timing” OR “meal frequency” OR “eating patterns” OR “meal patterns” OR “feeding behaviour” OR “meal regularity”) AND (“observational study” OR “prospective” OR “cohort” OR “cohorts” OR “longitudinal” OR “followup”).

Studies included had to investigate skipping breakfast as the exposure and the outcome had to be incident CVD, cardiovascular mortality, or all-cause death. The population of interest comprised adults without CVD at baseline. The results of the electronic database searches were first exported to Endnote and duplicates were removed. The title and abstract of each study were then screened. If the title or abstract seemed relevant, the full text of the record was evaluated. Only prospective cohort studies published in English were included. Conference abstracts, reviews and meta-analyses, editorials, non-English studies, studies with irrelevant exposures (e.g., combining skipping breakfast and late-night eating) or irrelevant outcomes, and study designs that could not estimate incidence (cross-sectional, case-control, and ecological studies) were excluded.

### 2.2. Data Extraction, Quality Assessment, and Synthesis

For each study, the following data were extracted: name of first author, year of publication, the country where the study was conducted, the cohort name (if any), duration of follow-up, baseline characteristics of the cohort (mean age, percentage of females), and total number of participants. In addition, we extracted information on exposure (skipping breakfast) definition, exposure assessment (questionnaire with or without validation, interviews), and data on risk estimates expressed as relative risk (RRs) or hazard ratios (HRs) with corresponding 95% confidence intervals (CIs), as well as the adjustment factors. Where available, we extracted risk estimates of composite CVD, as well as for specific conditions such as stroke and myocardial infarction. If studies defined skipping breakfast using frequencies rather than as a dichotomous exposure (yes vs. no), we selected the data comparing regular breakfast eating (every day) and never/rarely (0–2 days per week). Data were extracted by R.O. and cross-checked by A.O. All studies were read and assessed by two reviewers (R.O and A.O). There was 100% agreement between the two reviewers regarding the studies to be included or excluded. Each study’s quality was assessed against the Newcastle–Ottawa Scale [20]. Studies were ineligible for inclusion if they were rated below 5 on a scale from 0 to 9 following the checklist. The results of individual studies were pooled using random effects (DerSimonian and Laird) meta-analysis techniques. A leave-one-out sensitivity analysis was performed to examine the stability of the pooled estimate. The statistical evidence of between-study heterogeneity was examined using the Cochran’s Q test and the *Ι*^2^ statistic. *I*^2^ values of 25, 50, and 75% were considered to be low, moderate, and high degrees of heterogeneity, respectively. All analyses were performed using STATA version 15.0/IC (StataCorp, College Station, TX, USA). A *p*-value of <0.05 was considered statistically significant.

## 3. Results

### 3.1. Search Results and Study Characteristics

**Figure 1:**
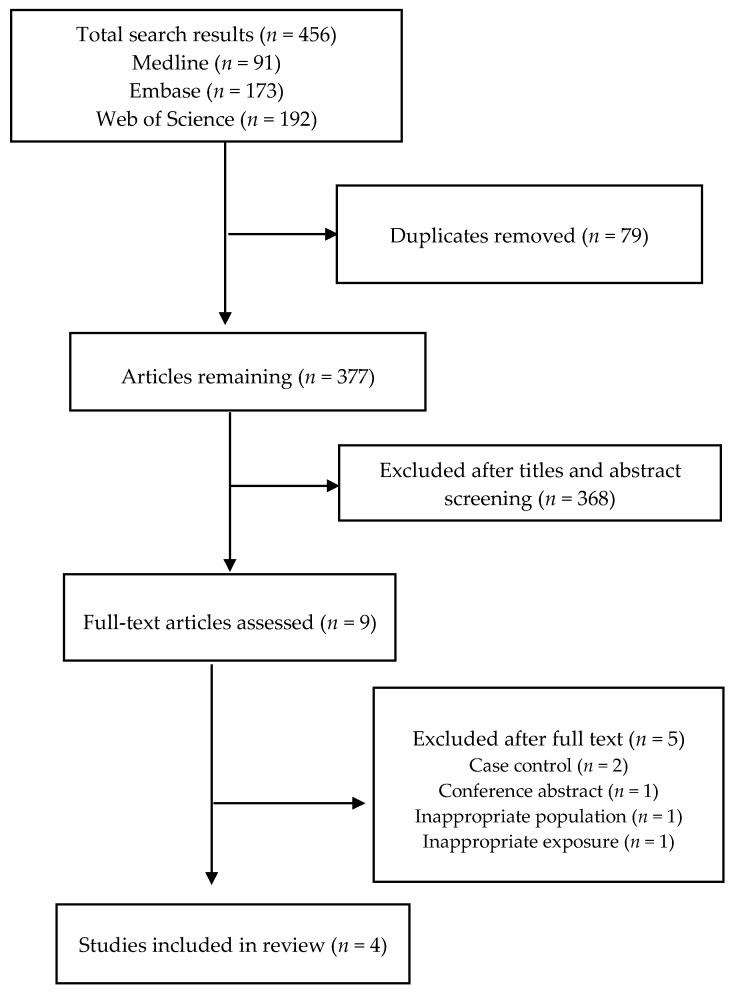
PRISMA flowchart of studies selection process.

The electronic database searches retrieved 456 citations. After the removal of duplicates and titles and abstract screening, nine articles were selected for full text evaluation, of which four were included in the final review (Figure 1) [21,22,23,24]. Reasons for exclusion were conference abstract (*n* = 1), case-control study (*n* = 2), inappropriate exposure (*n* = 1), and inappropriate population (*n* = 1). The included studies were from Japan (*n* = 2) and the United States (*n* = 2). The studies involved a total of 199,634 adults aged ≥40 years (48.5% female) without known CVD at baseline. The sample size in individual studies ranged from 6550 to 83,410 participants. The mean duration of follow-up was 17.4 (range 12.7–19.4) years (Table 1). Data were collected from participants via self-administered questionnaires in three studies, whilst one study used home-based interviews. No study was excluded on the basis of quality assessment against the Newcastle–Ottawa Scale.

### 3.2. Association of Skipping Breakfast with CVD Morbidity and Mortality

Cahill et al. [21] reported that in age-adjusted models, men who did not eat breakfast had a 33% increased risk of coronary heart disease (CHD) compared with men who did (risk ratio (RR) = 1.33, 95% CI 1.13–1.57). This risk was similar (RR 1.27, 95% CI 1.06–1.53) when also adjusted for diet, demographic, and activity factors, but was attenuated (RR 1.18, 95% CI 0.98–1.43) when further adjusted for body mass index (BMI), hypercholesterolemia, hypertension, and diabetes.

Kubota and colleagues [22] also reported an inverse association between the frequency of breakfast intake and the risks of total CVD, total stroke, and cerebral hemorrhage, after adjusting for dietary and lifestyle factors. Compared to people who consumed breakfast seven times per week, those who consumed breakfast zero to two times per week were 14% (hazard ratio (HR) 1.14, 1.01–1.27), 18% (HR 1.18, 1.04–1.34), and 36% (HR 1.36, 95% CI 1.10–1.70) more likely to develop CVD, stroke, and cerebral hemorrhage, respectively. However, the authors observed no significant association between the frequency of eating breakfast and the risk of CHD, although, they attributed this to the relatively low prevalence of CHD in the Japanese population, leading to the low power.

Using data from the National Health and Nutrition Examination Survey (NHANES), Rong et al. [23] reported that skipping breakfast was significantly associated with the risk of cardiovascular mortality, especially stroke-specific mortality, which persisted after adjusting for demographic, socioeconomic, dietary, and lifestyle factors, BMI, and cardiovascular risk factors. Specifically, compared to people who reported consuming breakfast every day, those reporting never consuming breakfast were 1.87 and 3.34 times more likely to die from CVD or stroke, respectively.

Based on data from the Japan Collaborative Cohort (JACC) Study for Evaluation of Cancer Risks, Yokoyama and colleagues [24] reported that skipping breakfast was associated with an increased risk of death from circulatory diseases in men (HR 1.42, 95% CI 1.02–2.02), but not women (HR 1.19, 95% CI 0.71–1.05), after adjusting for medical, sociodemographic, and lifestyle factors.

**Figure 2:**
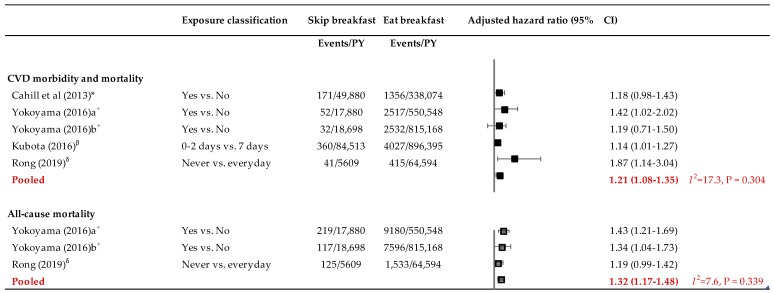
Forest plot of the association between breakfast skipping and cardiovascular disease morbidity and mortality and all-cause death. Covariates adjusted for in individual studies: * In addition to age, diet, demographic, and activity factors, this model was further adjusted for the body mass index (BMI) updated every 2 years (<18.5, 18.5–24.9, 25–29.9, ≥30 kg/m^2^, missing) as well as diabetes mellitus (yes/no), hypertension (yes/no), and hypercholesterolemia (yes/no), also updated every 2 years. ^+^ Adjusted for age and history of hypertension, history of diabetes mellitus, body mass index, smoking status, alcohol status, education level, physical activity, walking duration, sleep duration, marital status, and work schedule. ^β^ Adjusted for age, sex, ethanol, energy, and intake of vegetables, fruits, fish, soy, milk/dairy products, nuts, saturated fatty acid, dietary fiber, and sodium, as well as smoking status, leisure-time sports, sleep duration, perceived mental stress, living alone, physical labor, and public health center area. ^δ^ All models were adjusted for age, sex, race/ethnicity, marital status, family income level, smoking status, alcohol intake, physical activity, total energy intake, overall diet quality indicated by the Healthy Eating Index—2010, body mass index, hypertension, diabetes mellitus, and dyslipidemia. CI = confidence interval.

Overall, pooling data from the four included studies suggested that people who regularly skipped breakfast were 21% more likely to experience CVD or die from it than people who ate breakfast regularly (HR 1.21, 95% CI 1.08–1.35; *I*^2^ = 17.3%, *p* = 0.304) (Figure 2). A leave-one-out sensitivity analysis suggested a variation of the effect size from 1.17 to 1.25, which were all statistically significant towards an increased risk of CVD morbidity and mortality among people who regularly skipped breakfast compared to those who ate it regularly.

### 3.3. Association of Skipping Breakfast with All-Cause Mortality

Two prospective cohort studies reported the association between skipping breakfast and the risk of all-cause death. Rong et al. [23] found a non-significant increase in the risk of all-cause death among people who never ate breakfast compared to those who consumed breakfast every day (HR 1.19, 95% CI 0.99–1.42). Nonetheless, a Japanese study found that skipping breakfast was associated with increased risk of all-cause death in both males (HR 1.43, 95% CI 1.21–1.69) and females (HR 1.34, 95% CI 1.04–1.73) [24]. The pooled estimates from these two studies (3 data points) showed a 32% higher risk of death from all causes among people who regularly skipped breakfast compared to those who regularly consumed it (HR 1.32, 95% CI 1.17–1.48; *I*^2^ = 7.6%, *p* = 0.339). A leave-one-out sensitivity analysis suggested a variation of the effect size from 1.24 to 1.40, which were all statistically significant towards an increased risk of all-cause death among people who regularly skipped breakfast compared to those who ate it regularly.

## 4. Discussion

Nutrition plays an important role in the maintenance of overall health [25,26,27]. Recently, a statement from the American Heart Association (AHA) underscored the substantive role of meal timing and frequency in the prevention of CVDs [28].

In this study, we reviewed the evidence regarding the association between skipping breakfast and the prospective risk of cardiovascular morbidity and mortality and all-cause death. The magnitudes of association were variable across studies but overall suggested that people who skip breakfast may be at greater risk of experiencing adverse health outcomes compared to people who regularly consume breakfast. Specifically, we found that people who regularly skipped breakfast were about 21% more likely to suffer a CVD event or die from it, and 32% more likely to die from all causes than people who regularly ate breakfast.

Although our analysis focused on studies performed within CVD primary preventive settings, two studies that reported the effect of skipping breakfast on all-cause mortality in non-primary-preventive settings also showed that skipping breakfast alone or in combination with late night dinner was associated with increased risk of all-cause death [29,30]. Among patients with ST-segment elevation myocardial infarction, skipping breakfast concomitant with late night dinner was associated with 4–5-fold increased likelihood of death, reinfarction, and postinfarction angina within 30 days after hospital discharge [30].

Omitting breakfast has been associated with obesity, hypertension, diabetes, and atrial fibrillation, and may also impair serum lipids and postprandial insulin sensitivity [8,15,16,17,31,32]. Recently, skipping breakfast has also been shown to have an adverse effect on arterial stiffness and carotid atheromatic burden [33]. Furthermore, in mice, skipping breakfast has been shown to result in a disturbance of the phase peak of the clock gene, and an inability to entrain to the day/night cycle, thereby ultimately leading to disturbance of the circadian rhythm [24,34]. All these mechanisms may underlie the increased risk of cardiovascular morbidity and mortality among people who skip breakfast compared to those who regularly consume it. Nonetheless, studies suggest that eating breakfast may be a marker of lifestyle pattern [35,36,37]. For example, people who skip breakfast are more likely to smoke, have lower physical activity, low levels of total energy intake, eat dinner at irregular times and snack every day, or may have higher intakes of red and processed meat, appetizers, and alcohol during the rest of the day [37,38]. Skipping breakfast has also been related to sleep duration and shift work, both of which have been shown to increase the risk of CVD [14,39,40]. These factors may therefore potentially confound the association between skipping breakfast and the risk of cardiovascular morbidity and mortality as well as all-cause death.

While most studies attempted to adjust for potential confounders, data availability meant that there was significant variation in the confounders being adjusted for. Thus, residual confounding remains an issue [41] and should be considered when interpreting and comparing the findings of studies. For example, Kubota and colleagues [22] adjusted for sleep duration and perceived mental stress in their regression models, but such factors were not adjusted for in the analysis by Rong et al. [23]. It is known that short sleep duration is associated with increased risk of CHD mortality [42]. Moreover, Kubota and colleagues [22] argued that because obesity, hypertension, hypercholesterolemia, and diabetes mellitus could be mediators of the association between breakfast and CVD risk, they did not adjust for these in their regression analysis. However, these factors were included in the models presented by Rong et al. [23], Cahill et al. [21], and Yokoyama et al. [24]. These disparities in analytic framework may have contributed to the observed differences in the results. An equally important point also is that in nearly all the studies included in this review, the authors collected information on the frequency of breakfast intake only at baseline. Therefore, it is possible that participants might have changed their eating habits during the follow-up, which potentially introduces misclassification bias.

The finding of Yokoyama et al. [24] that skipping breakfast was associated with increased risk of death from circulatory diseases in men but not in women suggests possible gender differences in the effect of skipping breakfast. At the Experimental Biology meeting in Boston in 2015, Cahill and colleagues presented preliminary findings from the 56,593 women in the Nurses’ Health Study which showed that skipping breakfast was associated with increased risk of CHD in people aged <60 years but not in those aged 60 years and older [43]. Furthermore, a pioneering longitudinal study by Kaplan and colleagues found that skipping breakfast was associated with an increased risk of all-cause mortality in people aged ≥70 years but not in those aged less than 70 years [29]. Moreover, age and gender-related differences in the patterns of breakfast consumption have been observed [44,45]. Thus, future studies should explore in detail the potential gender and age-related differences in the association between skipping breakfast and the risk of adverse health outcomes.

It is important to also highlight that the included studies were from Japan and the US, two countries with very different cultural contexts and eating habits [46,47]. Landmark studies such as the NiHonSan study, which compared cardiovascular risk in Japanese men living in Japan, Hawaii, and California following post-World War II migration, illustrated the importance of environmental and dietary behavioral factors as contributors to cardiovascular risk, and found that living in a ‘Western’ environment was associated with increased CV risk [48]. The traditional Japanese breakfast of soup and side dishes containing soybean products, seafood, or vegetables [49] differs markedly from US breakfasts which are commonly high in refined grains and milk [11]. Regardless, due to the globalization of the food supply, some shifts away from traditional Japanese breakfasts have been observed among some populations in Japan [50,51].

Another issue relates to what constituted breakfast and how skipping breakfast was defined in the included studies. While some defined skipping breakfast on the basis of consumption, others based their definition on the basis of timing [21]. The definition of what constitutes breakfast is controversial [8,52]. In particular, there are concerns, for example, that using the time of day as a means of defining meals is restrictive to specific cultures and inappropriate for some population subgroups such as night or shift workers [14,28]. O’Neil and colleagues proposed a more unified definition of breakfast as “the first meal of the day that breaks the fast after the longest period of sleep, occurs within 2 to 3 h of awakening, and contains foods and beverages from at least one food group” [53], a definition that has been adopted by the AHA [28]. To improve the comparability of future studies, uniform definitions of both breakfast and skipping breakfast are required. Lastly, the included studies did not examine whether different types of foods or beverages eaten at breakfast have different effects on cardiovascular and overall health; this should be investigated in future studies.

## 5. Conclusions

Pooled data from a small number of published prospective cohort studies from the US and Japan suggest that skipping breakfast is associated with an increased risk of cardiovascular disease morbidity and mortality as well as all-cause death. However, the definition of skipping breakfast was highly heterogenous and residual confounding factors pose a challenge for the interpretation of available data. Large prospective studies that utilize consistent definitions of skipping breakfast and are conducted across different populations are needed to provide more robust evidence of the health effects of skipping breakfast.

## Figures and Tables

**Table 1:** Descriptive characteristics of included studies.

Author Details	Country of Study	Study (Cohort) Name	Sample Size	Age (Years)	% Female	Duration of Follow-Up (Years)	Breakfast Evaluation Method	Exposure (Breakfast) Definition	Outcome(s)
**Cahill et al., 2013 [21]**	US	Health Professionals Follow-Up Study	26,902	45–82	0.0	16.0	Questionnaire (self-administered)	Breakfast was defined as a positive response to any of the first three eating times (‘before breakfast’, ‘breakfast’, ‘between breakfast and lunch’)	Incident coronary heart disease (CHD); defined as non-fatal myocardial infarction (MI) or fatal CHD
**Kubota et al., 2016 [22]**	Japan	The Japan Public Health Center-Based Prospective (JPHC) study	82,772	45–74	53.3	12.7	Questionnaire (self-administered)	Participants were classified into the following four groups; those who had breakfast 0 to 2 (subjects with almost never, those with 1–3 times/month, and those with 1–2 times/week were combined because of the small number of those with 1–3 times/month or 1–2 times/week), 3–4, 5–6, or 7 (everyday) times/week. Those who had breakfast 7 times/week were regarded as the reference group.	Stroke and CHD (i.e., myocardial infarction and sudden cardiac death)
**Rong et al., 2019 [23]**	US	National Health and Nutrition Examination Survey III	6550	40–75	52.0	18.8	Home-based interviews	Participants were asked “How often do you eat breakfast?” during the household interview, and the possible answers included “every day,” “some days,” “rarely,” “never,” and “weekends only.” The frequency of breakfast eating was classified as “never,” “rarely,” “some days,” or “every day.”	Death from cardiovascular disease (CVD) (defined as heart disease or stroke; ICD codes: (I00–09, I11, I13, I20–51, I60–69), heart disease, stroke, or all-cause death.
**Yokoyama et al., 2016 [24]**	Japan	The Japan Collaborative Cohort Study (JACC) Study	83,410	40–79	59.1	19.4	Questionnaire (self-administered)	The type of breakfast consumed was assessed according to the following five categories: Japanese style, Western style, Chagayu style (tea rice gruel), no or nearly no breakfast eaten, or other. Participants were classified into two groups, as those who eat breakfast (including Japanese style, Western style, Chagayu style (tea rice gruel), and other) and those who skip breakfast (no or nearly no breakfast eaten).	Deaths from circulatory diseases (I00–I99) or all-cause death.
